# Mushroom Poisoning Mimicking Painless Progressive Jaundice: A Case Report with Review of the Literature

**DOI:** 10.7759/cureus.2436

**Published:** 2018-04-05

**Authors:** Abhilash Perisetti, Saikiran Raghavapuram, Abu Baker Sheikh, Rachana Yendala, Rubayat Rahman, Mohamed Shanshal, Kyaw Z Thein, Asif Farooq

**Affiliations:** 1 Department of Hospital Medicine, Texas Tech University Health Sciences Center, Lubbock, USA; 2 Division of Gastroenterology, University of Arkansas for Medical Sciences, Little Rock, USA; 3 Internal Medicine, University of New Mexico, Albuquerque, USA; 4 Hematology Oncology, Texas Tech University Health Sciences Center, Lubbock, USA; 5 Hospital Medicine, Texas Tech University Health Sciences Center, Lubbock, USA

**Keywords:** mushroom, poisoning, jaundince, pancreatitis

## Abstract

Mushroom poisoning is common in the United States. The severity of mushroom poisoning may vary, depending on the geographic location, the amount of toxin delivered, and the genetic characteristics of the mushroom. Though they could have varied presentation, early identification with careful history could be helpful in triage. We present a case of a 69-year-old female of false morel mushroom poisoning leading to hepatotoxicity with painless jaundice and biochemical pancreatitis.

## Introduction and background

Mushroom poisoning is common in the United States with 6000 exposures annually [1]. In some regions (the Rocky Mountain region and the Pacific Northwest) the reporting is quite extensive [2]. Of the various mushroom types, cyclopeptide containing mushrooms (Amatoxin, Phallotoxins) and Gyromitrin type cause liver toxicity [2]. Amanita poisoning has been reported to cause severe liver injury [3]. Seasonal variation might help in predicting the type of poisoning—Amanita species (death angel) occurring in fall and Gyromitra species (false morel) in spring and summer. Though they could have varied presentation, early identification with careful history could be helpful in triage.

Below, we report a case of false morel mushroom poisoning leading to hepatotoxicity with painless jaundice and biochemical pancreatitis.

## Review

Case

A 69-year-old Caucasian female with a history of atrial fibrillation (AF), hypertension and leg swelling reported to the local hospital due to nausea, vomiting and jaundice. She is a mushroom hunter for many years and reports consuming mushrooms before hospitalization. She takes warfarin, atenolol and furosemide. No history of alcohol or smoking or illicit drugs or herbal medications. Family and surgical history were unremarkable. She reported to the hospital with nausea, vomiting and painless jaundice. Her reviews of systems were negative. Physical exam showed alert, obese woman with jaundice, lower extremity pitting edema. Her vitals were significant for tachycardia (rate > 120 beats/min) with AF, blood pressure, respirations and temperature was within normal limits.

On admission, her lab results were significant for total bilirubin 13.6 mg/dl (N = 0.2-1 mg/dl) with direct bilirubin 9.7 (N = <0.2 mg/dl), AST-53 U/L (N = 0-31), ALT-57 U/L (N = 0-20), ALP 188 U/L (N = 35-104). International normalized ratio (INR) was elevated at 11. Serum lipase was 800 U/L (N = 73-393 U/L). Her complete blood count (CBC) with differential was unremarkable. Her creatinine was elevated at 1.5 mg/dl (baseline < 1). Her lactate was 7.5 mEq/L (N = 0.5-2.2 mEq/L). Viral hepatitis panel, immunological testing, lipid panel, muscle enzymes were normal. CA 19-9 was elevated at 71 U/ml (N-0-31). A workup for painless jaundice including ultrasound abdomen, computed tomography (CT) scan abdomen was negative for biliary ductal dilation but showed pancreatic haziness. A 2D cardiac echo showed decreased systolic function with ejection fraction (EF) of 21%. She was admitted to intensive care unit (ICU), resuscitated and her symptoms improved. Her bilirubin peaked at 16 mg/dl and trended down rapidly in two days and was 6.9 mg/dl during discharge; aspartate transaminase (AST) and alanine transaminase (ALT) were trending down. On further questioning, she acknowledges to consuming wild mushroom (looks like morels) hunted and given by his son in the beginning of summer, a day before presentation. She cooked the mushrooms and reports no symptoms in rest of the family. A probable diagnosis of mushroom poisoning was made with history, symptoms and exclusion of other causes.

Discussion

Mushroom poisoning is common in North America. They usually have symptoms of gastroenteritis, liver toxicity, and may cause seizures and methemoglobinemia [4,5]. Few cases in which ingestion of a toxic mushroom caused severe liver damage ultimately requiring organ transplantation [6]. Hepatotoxic mushrooms are few in number (Table 1).

**Table 1:** Hepatotoxic mushrooms.

Species	Amanita	Gyromitra	Lepiota	Others
Cases (n)	64	9	3	9

Most of the mushroom poisoning cases are secondary to Amanita species [2]. Gyromitra has been traditionally named false spring morel [7]. Most commonly, Gyromitra esculenta (false morel) is mistaken for the similar appearing Morchella esculenta (morel). False morels are irregularly shaped and wrinkled like the surface of the brain [8]. There was no case fatalities reported secondary to Gyromitra poisoning so far [2]. It usually accounts for less than 5% of human mushroom poisonings [2].

North American Mycological Association (NAMA) reported 27 cases of gyromitrin poisoning of which nine cases (33%) showed liver injury [2], however type of liver injury is unknown. Isolated cases reported elevated AST, ALT up to 431 and 472, respectively, and bilirubin of 3 mg/dl [8].

Above case shows unusual presentation of significant direct hyperbilirubinemia with minimal elevation of transaminases. Besides, a biochemical pancreatitis with elevated lipase and imaging was a rare association. Although microlithiasis could not be ruled out, improvement of the bilirubin on fluid resuscitation was significant. Reports of atrial fibrillation were reported with Gyromitrin but not specific [2]. In our case, there was worsening of atrial fibrillation with rapid rate which improved after two days of conservative therapy. Cardiac systolic dysfunction with low EF might be an effect of rapid AF noticed.

A review of the literature revealed Gyromitrin (acetaldehyde methylformylhydrazone) and its homologues are toxic compounds that convert in vivo into N-methyl-N-formylhydrazine (MFH), and then into N-methylhydrazine (MH) [9]. The toxicity of these chemicals, which are chiefly hepatotoxic and even carcinogenic, has been established through in vivo and in vitro experiments using animals, cell cultures and biochemical systems [10], a substance similar to the hydrazines in rocket fuel [10,11]. Carcinogenicity potential of gyromitra was identified in rats [9]. It is known to cause reversible enlargement of the liver in rats [12].

Literature review of the type of hepatotoxicity with mushroom poisoning is shown in Table 2.

**Table 2:** Literature review of type of hepatotoxicity with mushroom poisoning. AST: Aspartate transaminase; ALT: Alanine transaminase.

References	Patients (n)	Age (years)	AST (IU/L)	ALT (IU/L)	Total Bilirubin (mg/dl)	Outcome
Akman et al.	2	16, 4	3191– 4638	3761– 5148	12	Liver transplant (LT)
Garcia de la Fuente et al. [14]	1	1.5	14000	14000	4	LT
Tamme et al. [15]	1	52	16420	9402	4	LT
Mendez-Navarro et al.	2	62, 47	1290,2064	3900, 3146	21	Survived (S) Dead (D)
Trabulus and Altiparmak	144 (Amanita)		2500	2600	4.5	130 S 14 D
Mottram et al.	1 (Lepiota)	43	4200	2920	Unknown	S
Yardan et al.	4		>2000	>2000	Unknown	Unknown
Rogart et al.	1	63	2975	2130	34.7	D

## Conclusions

We report an unusual presentation of probable false mushroom poisoning presenting as painless jaundice with minimal AST, ALT elevation, biochemical pancreatitis, worsening AF and rapid improvement of symptoms after fluid resuscitation.

## References

[1] Bronstein AC, Spyker DA, Cantilena LR Jr, Green JL, Rumack BH, Giffin SL (2010). 2009 Annual Report of the American Association of Poison Control Centers' National Poison Data System (NPDS): 27th Annual Report. Clin Toxicol (Phila).

[2] Beug MW, Shaw M, Cochran KW (2006). Thirty-plus years of mushroom poisoning: summary of the approximately 2,000 reports in the NAMA case registry. McIlvainea.

[3] French LK, Hendrickson RG, Horowitz BZ (2011). Amanita phalloides poisoning. Clin Toxicol (Phila).

[4] Trabulus S, Altiparmak MR (2011). Clinical features and outcome of patients with amatoxin-containing mushroom poisoning. Clin Toxicol (Phila).

[5] Yardan T, Baydin A, Eden AO, Akdemir HU, Aygun D, Acar E, Arslan B (2010). Wild mushroom poisonings in the Middle Black Sea region in Turkey: analyses of 6 years. Hum Exp Toxicol.

[6] Skaare VK (1997). Mushroom poisoning: an indication for liver transplantation. J Transpl Coord.

[7] Flammer R (1985). Gyromitra syndrome: poisoning by the spring false morel (Article in German). Schweiz Rundsch Med Prax.

[8] Leathem AM, Dorran TJ (2007). Poisoning due to raw Gyromitra esculenta (false morels) west of the Rockies. CJEM.

[9] Slanina P, Cekan E, Halen B, Bergman K, Samuelsson R (1993). Toxicological studies of the false morel (Gyromitra esculenta): embryotoxicity of monomethylhydrazine in the rat. Food Additives Contaminants.

[10] Michelot D, Toth B (1991). Poisoning by Gyromitra esculenta--a review. J Appl Toxicol.

[11] Toth B (1995). Mushroom toxins and cancer (review). Int J Oncol.

[12] Braun R, Gerdes T, Steffen C, Netter KJ (1982). Influence of the mushroom poison gyromitrin on the lipids of rat liver (Article in German). Arzneimittelforschung.

[13] Akman SA, Cakir M, Baran M (2009). Liver transplantation for acute liver failure due to toxic agent ingestion in children. Pediatr Transplant.

[14] Garcia de la Fuente I, McLin VA, Rimensberger PC, Wildhaber BE (2011). Amanita poisoning and liver transplantation: do we have the right decision criteria?. J Pediatr Gastroenterol Nutr.

[15] Hallik M, Tamme K, Väli T, Starkopf J (2011). Successful liver transplantation after 21 days of hepatic coma. ASAIO J.

[16] Méndez-Navarro J, Ortiz-Olvera NX, Villegas-Ríos M (2011). Hepatotoxicity from ingestion of wild mushrooms of the genus Amanita section Phalloideae collected in Mexico City: two case reports. Ann Hepatol.

[17] Mottram AR, Lazio MP, Bryant SM (2010). Lepiota subincarnata J.E. Lange induced fulminant hepatic failure presenting with pancreatitis. J Med Toxicol.

[18] Rogart JN, Iyer A, Robert ME, Levy G, Strazzabosco M (2008). Type I autoimmune hepatitis presenting with acute liver failure in the setting of wild mushroom ingestion. J Clin Gastroenterol.

